# Influence of Quercetin in the Temporal Regulation of Redox Homeostasis in *Drosophila melanogaster*

**DOI:** 10.1093/jisesa/iex040

**Published:** 2017-04-28

**Authors:** Perumal Subramanian, Kanimozhi Kaliyamoorthy, Jaime Jacqueline Jayapalan, Puteri Shafinaz Abdul-Rahman, Onn Haji Hashim

**Affiliations:** 1 Department of Biochemistry and Biotechnology, Faculty of Science, Annamalai University, Chidambaram 608 002, Tamil Nadu, India (psub@rediffmail.com; kaniiamthebest@gmail.com); 3 University of Malaya Centre for Proteomics Research (UMCPR), Faculty of Medicine, University of Malaya, 50603 Kuala Lumpur, Malaysia (jaime_jacklyn@um.edu.my),; 4 Department of Molecular Medicine, Faculty of Medicine, University of Malaya, 50603 Kuala Lumpur, Malaysia (terisar@um.edu.my; onnhashim@um.edu.my)

**Keywords:** circadian, *Drosophila melanogaster*, oxidative stress, quercetin

## Abstract

Numerous biological processes are governed by the biological clock. Studies using *Drosophila melanogaster* (L.) are valuable that could be of importance for their effective applications on rodent studies. In this study, the beneficial role of quercetin (a flavonoid) on H_2_O_2_ induced stress in *D. melanogaster* was investigated. *D. melanogaster* flies were divided into four groups (group I – control, group II – H_2_O_2_ (acute exposure), group III – quercetin, and group IV – quercetin + H_2_O_2_ treated). Negative geotaxis assay, oxidative stress indicators (protein carbonyls, thiobarbituric reactive substances [TBARS]), and antioxidants (superoxide dismutase [SOD], catalase [CAT], glutathione-S-transferase [GST], glutathione peroxidase, and reduced glutathione [GSH]) were measured at 4 h intervals over 24 h and temporal expression of heat shock protein-70 (Hsp70), Upd1 (homolog of IL-6 in *Drosophila*), and nitric oxide synthase (Nos) was analyzed by Western blotting. Groups II and IV showed altered biochemical rhythms (compared with controls). Decreased mesor values of negative geotaxis, SOD, CAT, GST, and GSH were noticed in H_2_O_2_, increased mesor of oxidative stress indicators (TBARS and protein carbonyl content) and a reversibility of the rhythmic characteristics were conspicuous after quercetin treatment. The expression levels of Hsp70, Upd1, and Nos were noticeably maximum at 04:00. Significant elevation of expression by H_2_O_2_ was nearly normalized by quercetin treatment. The possible mechanism by which quercetin modulates oxidant–antioxidant imbalance under oxidative stress could be ascribed to the modulation of the rhythmic properties. Our results will be helpful to understand the molecular interlink between circadian rhythm and oxidative stress mechanism.

Temporal rhythms over a 24 h period in living organisms are essential to organize numerous behavioral, physiological, biochemical and endocrinological functions over day–night cycles. Circadian/daily rhythms of antioxidant enzymes and synthesis of other nonenzymatic antioxidants have been reported in numerous organisms. For instance, superoxide dismutase (SOD), glutathione peroxidase (GPx), glutathione reductase (GR), catalase (CAT) and the nonenzymatic antioxidant, reduced glutathione (GSH) show a diurnal pattern in a several tissues in mammals (Wilking et al. 2013, Mendez et al. 2016). Further, temporal oscillations in the expression of antioxidant genes suggest that defense in opposition to oxidative stress are circadian-gated (Sengupta et al. 2016). In fruit flies (Beaver et al. 2012) and mammals (Baydas et al. 2002), daily rhythms in the expression of GSH and in the activity of SOD, GPx, and GR have been observed (Hardeland et al. 2003). It is well-known that disruption of redox homeostasis represents a major aspect of pathological conditions in fruit fly (Feany and Bender 2000, Apidianakis and Rahme 2011, Zhao and Haddad 2011).

In *Drosophila melanogaster*, mortality due to acute oxidative stress exhibits a rhythm matching with the light–dark cycle (LD) and a loss in rhythmicity in constant light condition (LL) was noticed (Krishnan et al. 2008). Furthermore, the null mutation in *per*, which abolishes clock function, augments the vulnerability to acute oxidative stress with no significant rhythmicity; this susceptibility to oxidative stress is recovered in flies with rescued *per* function (Krishnan et al. 2008) indicating a main role of biological clock on the susceptibility to an oxidative challenge. Moreover, *cry^b^* mutant flies with a defect in the circadian photoreception do not exhibit rhythmicity in enzymatic antioxidants (Subramanian et al. 2014).

The fruit fly, *D. melanogaster* is a well-suited model system to study biological timing system and has been used extensively for a long time to unravel the generation and regulation of circadian rhythms. *D. melanogaster* has also been widely used to comprehend the pathophysiology of many human diseases and to assess the therapeutic potential of phytochemicals (Girish and Muralidhara 2012) owing to very few ethical concerns and approval of this model system by European Centre for the Validation of Alternative Methods (ECVAM; Festing et al. 1998).

Quercetin (a penta hydroxyl flavone) is a plant-derived bioflavonoid (from tomatoes and onions) which is recently gaining scientific interest for its antioxidant, free radical scavenging, anti-angiogenesis, antitumor, and anti-inflammatory properties (Boesch-Saadatmandi et al. 2012, Kobori et al. 2015, Zhao et al. 2016). Quercetin at the dose used in this study (0.1%) was not found to be toxic in flies (Sotibran et al. 2011). This bioflavonoid exhibits therapeutic potential in various ailments like cancer, coronary artery, asthma and Alzheimer disease (Boesch-Saadatmandi et al. 2012, Kobori et al. 2015, Zhao et al. 2016). Additional clinical uses include treatment of inflammatory conditions like gout, pancreatitis and prostatitis (Hollman and Katan 1997). It has been extensively studied for its gastroprotective effects, anti-obesity action, immune booster, reducing risk of cataract, and reduction of diabetic complications (Gupta et al. 2016). Quercetin has exhibited the capability to avert the oxidative damage to low-density lipoproteins (LDL) by quenching free radicals and chelating metal ions. Consequently, quercetin could assist in the defense against several diseases, for instance, cancer, diabetes and atherosclerosis (Murota and Terao 2003). However, its protective influences on the temporal pattern of redox homeostasis have not been investigated so far.

Stress response and antioxidant defense coordination involve stress proteins (coined as heat shock proteins; Hsps) and antioxidants (enzymatic and nonenzymatic) as the primary defending responses that are well-conserved from flies to humans (Lindquist and Craig 1988). Amongst the stress genes family, Hsp70 is an exceedingly conserved gene and the first to be studied in *Drosophila* (Feder et al. 1992). Several investigations showed the association between Hsp70 synthesis and numerous several stress factors in the fly (Singh et al. 2009). For instance, the larvae of *D. melanogaster* fed on a diet with silver nanoparticles (Ag NPs; 50 and 100 μg/ml) showed upregulation of Hsp70 and elevated oxidative stress in *D. melanogaster* (Ahamed et al. 2010).

The homolog of mammalian IL-6 in *Drosophila* is *Upd1* (*unpaired*; Panayidou and Apidianakis 2013) and is known to be induced under stress conditions (Apidianakis and Rahme 2011). In *Drosophila* there is one form of nitric oxide synthase (Nos) coded by the gene *foxd* un-like three forms of NOS in mammals (*dNos*; Stasiv et al. 2001, Lacin et al. 2014). NO and its intermediate products can react with hydrogen peroxide (H_2_O_2_), superoxide anion (^•^O2^−^), or oxygen (O_2_), correspondingly, forming the potent cytotoxic peroxynitrite (ONOO^−^; Ignarro 1999). Nos oxidizes arginine to produce NO and citrulline (Regulski et al. 2004) and NO acts via cGMP signaling in neurons to stimulate cell death (Wang et al. 2015).

Female flies exhibited a decline in reproductive output and decreased survival while males were unaffected when the adverse effects of tea polyphenols were examined (Lopez et al. 2016) in the fruit fly. The levels of protein carbonyls and lipid peroxides, as well as activities of antioxidants (SOD and CAT) are known to vary depending on the gender of the fly (Rovenko et al. 2015). Since the reproductive activities of females interfered with our experimental protocols male flies were chosen for this investigation in order to compare with previous studies. Rodriguez et al. (2013) had reported the circadian control of 237 robust cycling mRNAs in adult fly heads. Further, organ-based variations in oxidative stress have been reported (Davies et al. 2012, Piegholdt et al. 2016). Different levels of reactive oxygen species (ROS) and hydroperoxides have been reported in head and body of the flies treated with acrylamide (Prasad and Muralidhara 2012). Acrylamide was known to induce differential increase in ROS levels in head (44%) and body (48%). Similarly, the hydroperoxide levels were also variably increased in head (37%) and body regions (48%). While malondialdehyde content (an indicator of lipid peroxidation) was slightly (17%) increased in body, they are markedly elevated (26%) in head (Prasad and Muralidhara 2012, 2014). Furthermore, the protein carbonyl content, GSH, and antioxidant enzymes (SOD and CAT) were found to show different levels of response to acrylamide induced oxidative stress in head and body of flies (Prasad and Muralidhara 2012, 2014). However, so far, temporal regulation of oxidative stress in head and body regions has not been investigated. Hence, we considered the head and body parts of the fly to assess the temporal regulation of organ-dependent oxidative stress. We also examined the hypothesis that administration of quercetin modulates indices of redox homeostasis, expression levels of Hsp70, Upd1 and Nos under oxidative stress (owing to acute H_2_O_2_ treatment) and reversibility of rhythms by quercetin.

## Materials and Methods

### 

#### System and Treatment

Wild type (Canton S) of *D. melanogaster* was used in the present study and the flies were maintained at 20 ± 2°C and reared on a diet medium (one unit of medium is 360 ml containing agar – 2.5 g, maize powder – 17 g, sucrose – 15 g, yeast tablets – 6 g, nepagin (antifungal agent) – 1 g and propionic acid – 1 ml) under 12:12 (L:D) h condition (lights on – 06:00 and lights off – 18:00). The flies were divided into four groups (group – I control, group – II hydrogen peroxide (100 µl, acute treatment), group – III quercetin (0.1%; administered through media), and group – IV quercetin + hydrogen peroxide treated). Acute exposure to hydrogen peroxide was performed as described previously (Subramanian et al. 2014) by exposing 5-day-old WT flies to 88 µM H_2_O_2_ (in 1% sucrose solution) after transferring them into vials containing filter paper moistened with 100 µl of H_2_O_2_ for 4 h prior to being placed into the regular culture vial; the H_2_O_2_ treatment causes 10% mortality within 24–48 h following exposure. The analyzes were carried out on the 3rd day.

#### Assays of Markers of Oxidative Stress

Adult male flies (*n* = 25) were collected at 4 h time intervals (00:00, 04:00, 08:00, 12:00, 16:00, 20:00 and 00:00) over the 24 h period and used for negative geotaxis assay and biochemical estimations. Immediately after collection, the head and body tissues of the fly were dissected under the microscope (10×). The tissue homogenate (1.5 ml) of head and body was prepared in eppendorf tubes using tissue homogenizer (Thomas Scientific, Swedesboro, NJ, USA) using 0.1 M sodium phosphate buffer (pH 7.5) and centrifuged (5,000 rpm for 15 min) at 4 °C and the supernatant was used for biochemical estimations.

Negative geotaxis assay was performed as described earlier (Feany and Bender 2000, Subramanian et al. 2014). Assays of protein carbonyl content (Levine et al. 1990), TBARS (Ohkawa et al. 1979), SOD, CAT (Aebi 1984), GPx (Rotruck et al. 1973), and GSH (Hissin and Hilf 1976) were performed in head and body tissue homogenates.

#### Temporal Oscillations

Time series of the oscillation (estimates of acrophase, amplitude and mesor by cosinor analysis) was done as proposed by Halberg et al. (1977) by using “cosinorwin” software (Cosinor 2.4, SEPTMR, Tucows Inc., Toronto, Canada) and cosine fitted curves were plotted as described previously (Subramanian et al. 2014), by using the equation, (*Y*_ti_ = *M *+* A* cos (*ωt* ‒ *ϕ*) where *Y*_ti_ – cosine function at the time point, *M* – Mesor, *A* – amplitude, *t* – time and *ϕ* – acrophase; Nelson et al., 1979). The acrophase (*ϕ*) is the estimate of peak time of the variable investigated. The amplitude (*A*) refers to half of the total rhythmic variation over 24 h period and mesor (*M*) is the rhythm adjusted mean (arithmetic mean of equidistant data covering a 24 h period). The acrophase is denoted in h and amplitude and mesor are denoted in the unit of the variable studied. Three replicates were performed and mean ± SEM values were represented (Table 1). Values of *r* and *P* of the oscillation were calculated by using “cosinorwin” software programme (Expert Soft Technologie Laboratory of Applied Statistics and Biomedical Computing, Chemic de la Birotte, Esvres, France) as described previously and a *P* value of <0.01 was deemed significant (Subramanian et al. 2014). The values of *r* and *P* for all the groups are mentioned in Table 1.

**Table 1.** The circadian characteristics (acrophase [*ϕ*], amplitude [*A*], and mesor [*M*]) of negative geotaxis and indices of redox homeostasis in head and body parts of *D. melanogaster*

Parameter	Groups	Acrophase (*ϕ*) (h)	Amplitude (*A*)	Mesor (*M*)	*r*-Value	*P*-value
Negative geotaxis	Control	6.16 ± 0.2	3.1 ± 0.4	24.90 ± 3.2	0.98	*P* < 0.01^dr^
	H_2_O_2_	7.51 ± 0.3	1.5 ± 0.1	22.90 ± 4.7	0.46	*P* < 0.05^ns^
	Quercetin	4.37 ± 0.4	2.8 ± 0.3	24.50 ± 2.4	0.55	*P* < 0.0005^dr^
	Quercetin + H_2_O_2_	8.80 ± 0.5	2.7 ± 0.3	23.90 ± 3.1	0.63	*P* < 0.01^dr^
Protein carbonyl content (head)	Control	17.17 ± 0.09	1.2 ± 0.4	3.8 ± 0.9	0.88	*P* <0.01^dr^
	H_2_O_2_	16.28 ± 0.3	0.2 ± 0.09	4.4 ± 0.2	0.45	*P* < 0.01^dr^
	Quercetin	17.23 ± 0.2	1.0 ± 0.2	2.6 ± 0.3	0.97	*P* < 0.01^dr^
	Quercetin + H_2_O_2_	4.50 ± 0.09	0.8 ± 0.09	3.5 ± 0.6	0.69	*P* < 0.01^dr^
Protein carbonyl content (body)	Control	22.41 ± 0.5	1.6 ± 0.2	2.4 ± 0.09	0.80	*P* < 0.01^dr^
	H_2_O_2_	18.29 ± 0.4	0.5 ± 0.05	4.5 ± 0.3	0.35	*P* < 0.01^dr^
	Quercetin	23.13 ± 0.08	1.3 ± 0.2	2.3 ± 0.08	0.93	*P* < 0.01^dr^
	Quercetin + H_2_O_2_	18.18 ± 0.4	0.9 ± 0.08	3.6 ± 0.4	0.97	*P* < 0.01^dr^
TBARS (head)	Control	8.56 ± 0.6	1.7 ± 0.1	8.3 ± 1.2	0.65	*P* < 0.01^dr^
	H_2_O_2_	7.43 ± 0.1	0.3 ± 0.08	14.8 ± 2.3	0.43	*P* < 0.01^dr^
	Quercetin	8.55 ± 0.3	0.8 ± 0.1	8.5 ± 1.1	0.76	*P* < 0.01^dr^
	Quercetin + H_2_O_2_	7.20 ± 0.7	1.3 ± 0.5	7.5 ± 0.09	0.62	*P* < 0.01^dr^
TBARS (body)	Control	8.23 ± 0.6	2.4 ± 0.8	9.4 ± 1.2	0.91	*P* < 0.01^dr^
	H_2_O_2_	5.55 ± 0.2	0.6 ± 0.09	16.3 ± 2.4	0.49	*P* < 0.01^dr^
	Quercetin	13.52 ± 0.5	2.1 ± 0.4	10.0 ± 1.3	0.76	*P* < 0.005^dr^
	Quercetin + H_2_O_2_	6.69 ± 0.01	2.1 ± 0.5	7.6 ± 0.9	0.81	*P* < 0.01^dr^
SOD (head)	Control	15.56 ± 0.3	2.1 ± 0.4	15.4 ± 2.5	0.65	*P* < 0.01^dr^
	H_2_O_2_	13.48 ± 0.4	0.2 ± 0.08	9.7 ± 1.6	0.25	*P* < 0.05^ns^
	Quercetin	15.41 ± 0.09	2.1 ± 0.6	14.2 ± 1.6	0.55	*P* < 0.001^dr^
	Quercetin + H_2_O_2_	12.12 ± 0.5	2.0 ± 0.8	10.9 ± 1.1	0.69	*P* < 0.01^dr^
SOD (body)	Control	14.54 ± 0.6	3.4 ± 0.9	15.3 ± 2.1	0.66	*P* < 0.01^dr^
	H_2_O_2_	14.27 ± 0.1	0.8 ± 0.06	9.9 ± 0.09	0.37	*P* < 0.01^dr^
	Quercetin	15.46 ± 0.6	2.9 ± 1.0	14.9 ± 2.0	0.56	*P* < 0.01^dr^
	Quercetin + H_2_O_2_	12.55 ± 0.3	3.1 ± 1.1	11.5 ± 0.9	0.55	*P* < 0.01^dr^
CAT (head)	Control	13.80 ± 0.09	3.3 ± 1.0	142.1 ± 12.8	0.86	*P* < 0.01^dr^
	H_2_O_2_	9.28 ± 0.4	0.4 ± 0.08	97.7 ± 6.9	0.41	*P* < 0.05^ns^
	Quercetin	13.11 ± 0.2	3.1 ± 0.9	131.4 ± 7.2	0.84	*P* < 0.01^dr^
	Quercetin + H_2_O_2_	10.17 ± 0.7	2.9 ± 0.9	89.9 ± 5.2	0.69	*P* < 0.01^dr^
CAT (body)	Control	3.36 ± 0.08	3.1 ± 0.8	95.8 ± 4.6	0.56	*P* < 0.01^dr^
	H_2_O_2_	13.57 ± 0.2	1.9 ± 0.7	82.7 ± 2.1	0.32	*P* < 0.01^dr^
	Quercetin	11.42 ± 0.6	2.8 ± 0.7	127.8 ± 9.6	0.97	*P* < 0.01^dr^
	Quercetin + H_2_O_2_	9.59 ± 0.09	2.3 ± 0.6	69.7 ± 3.1	0.61	*P* < 0.01^dr^
GST (head)	Control	18.25 ± 0.2	1.1 ± 0.08	15.07 ± 0.9	0.95	*P* < 0.01^dr^
	H_2_O_2_	15.02 ± 0.09	0.4 ± 0.03	11.3 ± 1.0	0.41	*P* < 0.02^ns^
	Quercetin	15.32 ± 0.3	0.8 ± 0.05	13.6 ± 1.7	0.65	*P* < 0.01^dr^
	Quercetin + H_2_O_2_	14.13 ± 0.2	0.9 ± 0.03	12.9 ± 1.4	0.51	*P* < 0.01^dr^
GST (body)	Control	17.20 ± 0.3	0.9 ± 0.03	14.2 ± 1.1	0.70	*P* < 0.01^dr^
	H_2_O_2_	18.04 ± 0.2	0.3 ± 0.01	10.67 ± 0.9	0.38	*p*<0.01^dr^
	Quercetin	15.49 ± 0.4	0.8 ± 0.06	14.9 ± 08	0.66	*P* < 0.01^dr^
	Quercetin + H_2_O_2_	16.70 ± 0.6	0.7 ± 0.07	13.3 ± 1.0	0.55	*P* < 0.01^dr^
GPx (head)	Control	10.24 ± 0.2	1.2 ± 0.05	8.3 ± 0.8	0.73	*P* < 0.01^dr^
	H_2_O_2_	8.4 ± 0.09	0.5 ± 0.05	5.3 ± 0.09	0.44	*P* < 0.05^ns^
	Quercetin	9.13 ± 0.3	0.9 ± 0.07	7.5 ± 0.08	0.88	*P* < 0.005^dr^
	Quercetin + H_2_O_2_	7.23 ± 0.7	0.8 ± 0.05	7.1 ± 0.07	0.62	*P* < 0.01^dr^
GPx (body)	Control	18.50 ± 0.3	0.9 ± 0.05	14.6 ± 0.09	0.87	*P* < 0.01^dr^
	H_2_O_2_	12.39 ± 0.2	0.3 ± 0.02	10.4 ± 0.9	0.21	*P* < 0.02^ns^
	Quercetin	3.30 ± 0.4	0.8 ± 0.02	12.5 ± 1.1	0.66	*P* < 0.01^dr^
	Quercetin + H_2_O_2_	14.14 ± 0.4	0.7 ± 0.01	9.8 ± 0.08	0.58	*P* < 0.01^dr^
GSH (head)	Control	11.44 ± 0.3	1.6 ± 0.09	13.3 ± 0.9	0.96	*P* < 0.01^dr^
	H_2_O_2_	11.60 ± 0.2	0.6 ± 0.06	8.7 ± 0.5	0.33	*P* < 0.05^ns^
	Quercetin	11.35 ± 0.5	1.3 ± 0.1	11.6 ± 0.6	0.98	*P* < 0.01^dr^
	Quercetin + H_2_O_2_	10.13 ± 0.08	1.2 ± 0.3	10.6 ± 0.4	0.66	*P* < 0.01^dr^
GSH (body)	Control	12.15 ± 0.09	1.0 ± 0.09	12.9 ± 0.6	0.90	*P* < 0.01^dr^
	H_2_O_2_	9.50 ± 0.5	0.4 ± 0.06	9.4 ± 0.6	0.46	*P* < 0.01^dr^
	Quercetin	11.38 ± 0.6	1.0 ± 0.05	12.5 ± 0.4	0.55	*P* < 0.01^dr^
	Quercetin + H_2_O_2_	11.54 ± 0.4	1.0 ± 0.07	11.8 ± 0.7	0.99	*P* < 0.01^dr^

*n* = 25 (mean ± SEM).

Acrophase (*ϕ*) (h).

Amplitude (*A*).

Mesor (*M*).

dr – detectable rhythmicity.

ns – no significant rhythmicity.

#### Temporal Expression of Hsp70, Upd1, and Nos

Adult male flies (*n* = 20) collected at 00:00, 04:00, 08:00, 12:00, 16:00 and 20:00 were used for the assessment of expressions of Hsp70, Upd1, and Nos by Western blot analysis. The extracted protein (50 μg) was mixed with 2× sample buffer, boiled for 5 min and was run on 12% SDS-PAGE gel for 2.5 h and electro transferred to a PVDF membrane (Millipore, Germany) for 1 h. The membrane was then treated with blocking buffer for 2 h. After 2 h, the blocked membranes were incubated with respective antibodies of Hsp70, Upd1, and Nos (GENEI, Bangalore, India; 1:1,000) for 6 h. After primary antibody incubation, the membranes were washed thrice with blocking buffer for 10 min subsequently. Membranes were incubated with horseradish peroxidase-conjugated IgG antibody (1:5,000). Subsequently the membrane was washed with 1× TTBS and TBS, and was developed using ECL (Pierce, Rockford, USA). The bands were visualized in Chemi Doc Imaging System (Bio-Rad, USA). Immunoblot for β-actin was used as an internal control. The expression level of proteins in all groups was statistically analyzed by ANOVA (two way analysis) at respective time points.

## Results

### 

The parameters showed temporal variations over 24 h period. In general, at daytime the amplitude (negative geotaxis, TBARS, SOD, CAT, GPx, and GSH) tend to increase and at night time the values tend to decrease although the acrophase did not follow this generalized pattern. Furthermore, protein carbonyl and GST (in head) tend to show low value during daytime. CAT (in head) and GSH (in body) showed narrower amplitude differences over day and night.

#### Negative Geotaxis Assay

The temporal characteristics (acrophase, amplitude, and mesor), *r* and *P* values indicate detectable rhythmicity or insignificant rhythmic variations in different groups (Table 1). The 24 h pattern of negative geotaxis assay revealed insignificant rhythmicity in H_2_O_2_ treated flies and an alteration in group III. Advanced acrophase and elevated mesor were found in group IV (compared with group III). Amplitude was decreased in H_2_O_2_ treated flies than control and other experimental groups (Fig. 1).

**Fig. 1.**
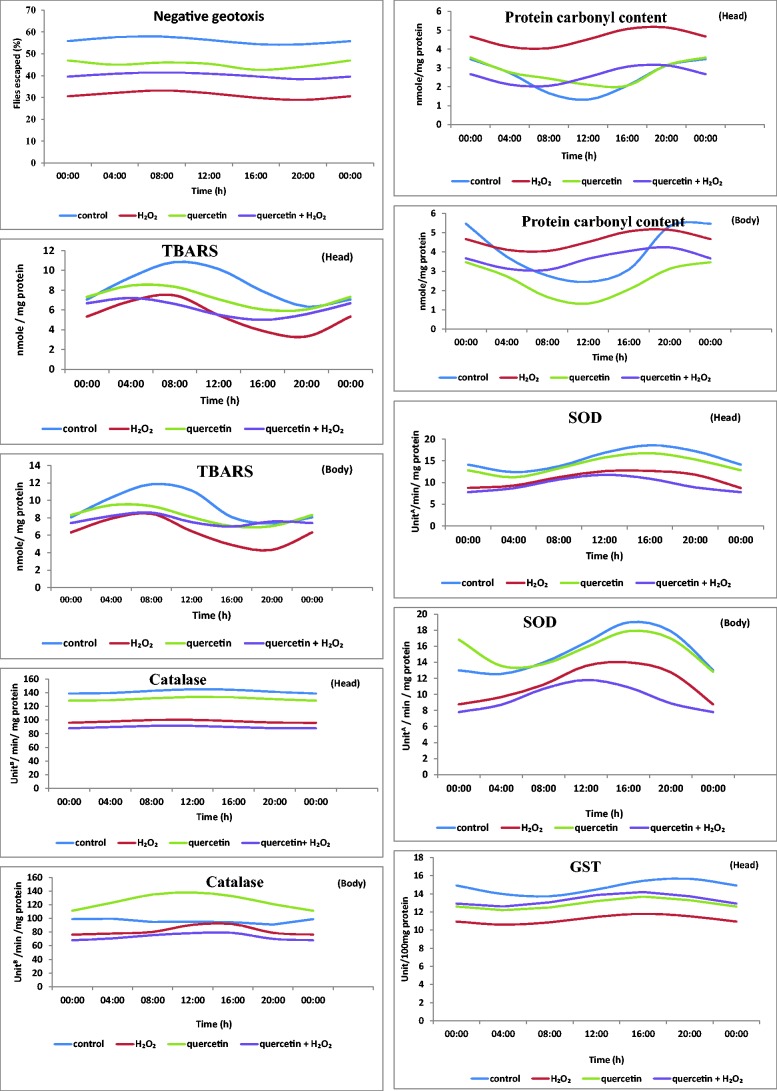

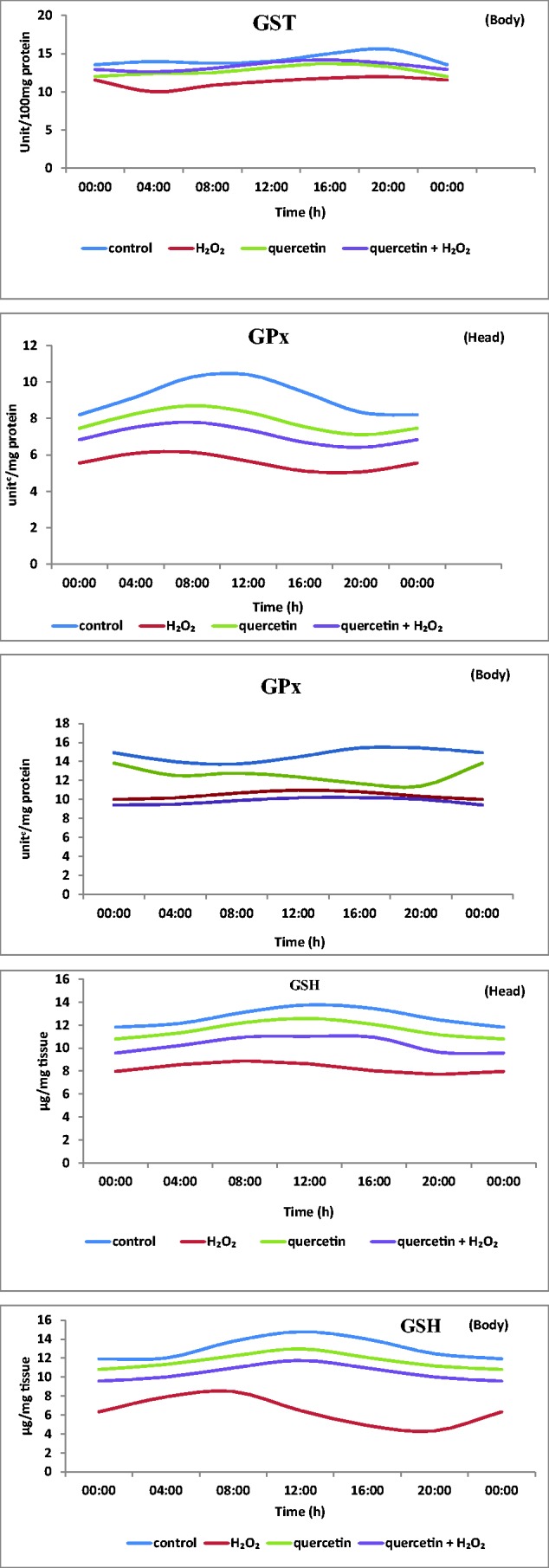
Representative cosine fitted curves of 24 h oscillations of negative geotaxis, protein carbonyl, TBARS, SOD, CAT, GST, GPx and GSH in head and body regions in control and experimental groups of flies.

**Fig. 1.**
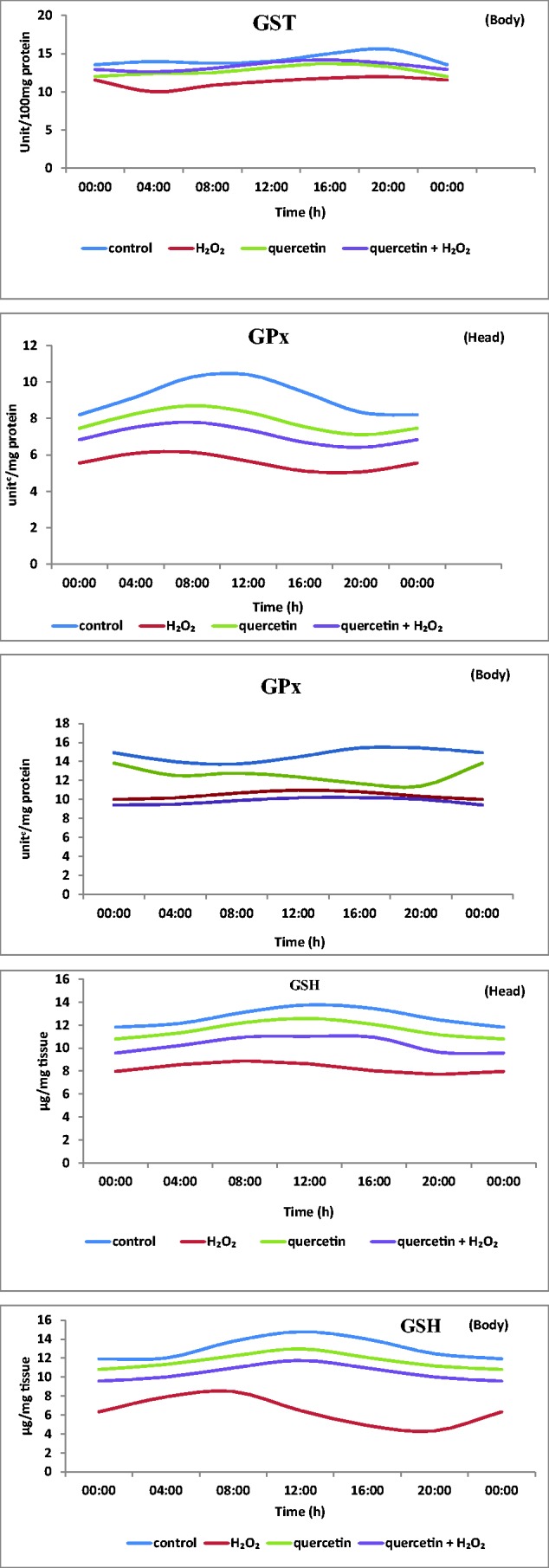
Continued.

#### Protein Carbonyl Content

The temporal patterns of protein carbonyl content revealed detectable rhythmicity and it was found to be disturbed in group II flies in head. Acrophase was advanced in H_2_O_2_ treated flies and advances in groups III and IV were noticed than control. Amplitude values were elevated in groups III and IV and decreased in group II. Temporal oscillation in body of the flies showed different acrophases of protein carbonyl content at 22.41 – group I, 18.29 – group II, 23.13 – group III, and 18.18 – group IV flies. Mesor level was increased in group II (compared with control; Fig. 1, Table 1).

#### Thiobarbituric Acid Reactive Substances (TBARS)

Detectable rhythmicity of TBARS in heads was noticed in all groups of flies. However, advanced acrophase and increased mesor values of TBARS was noticed in groups II and III when compared with control (Fig. 1). Amplitude values were increased in groups III and IV and decreased in group II. Elevated mesor values and advanced acrophase were found in H_2_O_2_ treated (body) flies. Delayed acrophase and decreased mesor values were found in group IV; amplitude value showed decrease in group II and a decrease in groups III and IV (Fig. 1, Table 1).

#### SOD

The temporal pattern of SOD was detectable in groups I, III and IV. Detectable rhythmicity was not noticed in group II flies in head. Acrophase was delayed in group II and advanced in group IV compared with control. Mesor was decreased in group II and increased in groups III and IV. The acrophase of SOD (in body) was at 14.56 in group I; whereas in groups II, III, and IV the values were maximum at 14.27, 15.46, and 12.55 respectively. Mesor was decreased in group II and increased in groups III and IV (Fig. 1, Table 1).

#### CAT

The detectable rhythm of CAT was absent in H_2_O_2_ treated flies (head). Acrophase was delayed in group II and advanced in groups III and IV. Mesor was decreased in group II and increased in groups II and IV. The peak activity of CAT (in body) was observed at 3.38 in group I; whereas in groups II, III and IV flies the maximum values lied at 13.47, 11.42, and 10:03 respectively. Increased mesor level in groups I, III and IV whereas decreased value in group II was noticed (Fig. 1, Table 1).

#### Glutathione-S-Transferase (GST)

Acrophase of GST (head) in groups I–IV was noticed at 18.21, 15.02, 15.32, and 14.13 respectively. Detectable rhythmicity was absent in group II (head). Acrophase was advanced at 18.04, 15.49, and 16.70 in groups II, III and IV (body) as compared to control. Amplitude was decreased in group II and was increased in groups III and IV (head and body). Mesor was decreased in group II and increased in groups III and IV (head and body) compared to control (Fig. 1, Table 1).

#### GPx

The temporal pattern of GPx revealed a detectable rhythmicity in groups I, III and IV and was not detectable (*P *< 0.05) in group II (head). Amplitude value was increased in groups III and IV and decreased in group II (head and body). Mesor was increased in groups III and IV as compared with group II (head and body; Fig. 1, Table 1).

#### GSH

The acrophase of GSH (head) was found to be at 11.44 in group I and at 11.06, 11.35, and 10.13 respectively from groups II to IV. Maximum values over 24-h period lied at 12.09 (group I), 9.04 (group II), 12.9 (group III), and 11.08 (group IV; in body). Decreased mesor in group II and increase in groups III and IV were noticed (body). Mesor level was increased in groups III and IV (Fig. 1, Table 1).

#### Temporal Expression of Hsp70, Upd1, and Nos

**Fig. 2.**
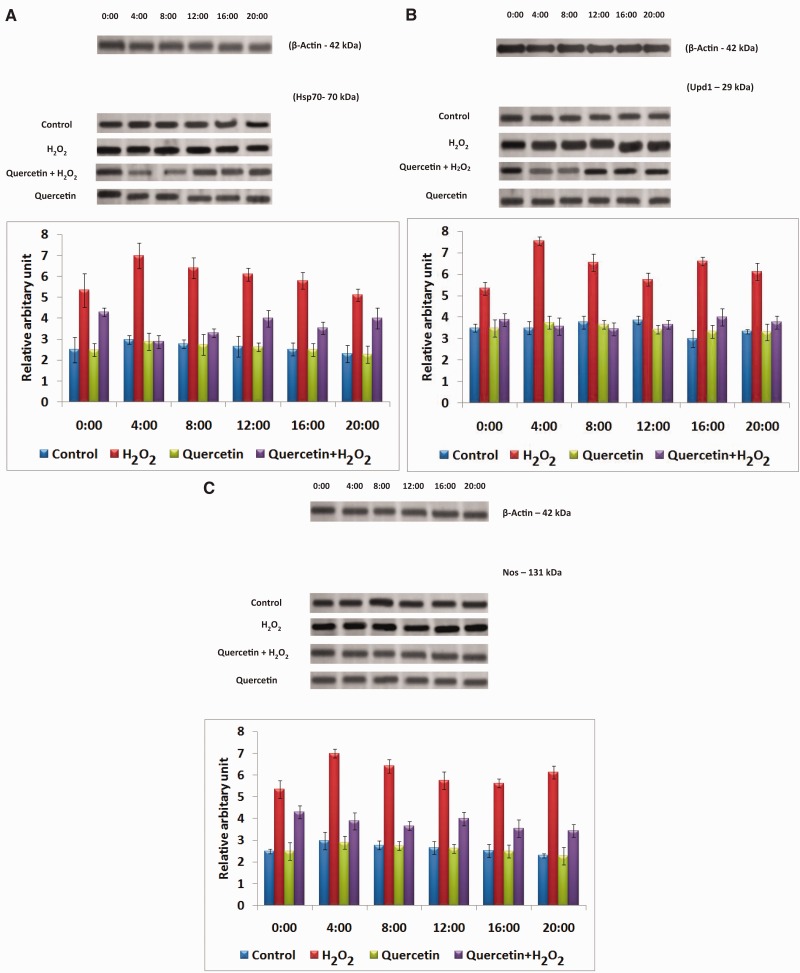
Temporal expression pattern and plot of expression level of Hsp70 (A), Upd1 (B), and Nos (C) in all the groups of *D. melanogaster* (*F* = 6.5; df = 18; *P *< 0.01 – H_2_O_2_ group compared with control; *F* = 7.2; df = 18; *P* < 0.01 – quercetin + H_2_O_2_ group compared with H_2_O_2_ group).

The expression levels of Hsp70, Upd1, and Nos showed noticeable variations over 24-h period and maximum level of expression was observed at 04:00. The *F*, df and *P* values are given in Figure 2. In H_2_O_2_ treated flies, significantly elevated expression of Hsp70, Upd1, and Nos was noticed (compared to control) and H_2_O_2_ + quercetin treatment caused notable decrement in the expression (compared to H_2_O_2_ treated) at all time points studied (Fig. 2).

## Discussion

The biological clock temporally systematizes numerous cellular processes relative to one another. Circadian activity-rest rhythm entails temporal variations in ROS amounts that are formed in excess during activity period and diminished metabolic activities during rest (Langmesser and Albrecht 2006) under normal conditions. As *D. melanogaster* is a day active insect, high levels of metabolic and locomotor activities might increase ROS during light period might be responsible for the augmented levels of TABRS and subsequent elevation of most of the antioxidant enzymes during day time.

Genomic microarray studies have indicated that rhythmic expression of genes is imperative for rhythmic metabolic rates and stress resistance in *Drosophila* (McDonald and Rosbash 2001, Ceriani et al. 2002). Temporal regulation of free radical damage has been documented in *D. melanogaster* (Coto-Montes and Hardeland et al. 1999) and there is a circadian component to the fly’s susceptibility to oxidative stress. This temporal variation could protect the fly from augmented levels of free radicals, by sustaining rhythmic redox homeostasis, and ultimately from the injury to the cellular constituents (Hardeland et al. 2003, Kondratov 2007). More importantly, the conserved biological pathways, including similarities between reproductive and developmental genes and hormones in flies and humans, make flies a valid model organism to evaluate drug-induced adverse effects (Avanesian et al. 2009, Kislukhin et al. 2013). Wild type flies experience lower mortality rates when exposed to exogenous H_2_O_2_ in the night time compared to the flies subjected to a condition of constant light, which disrupts the circadian clockwork (Krishnan et al. 2008).

The flavonol, quercetin (3,5,7,3′,4′-pentahydroxyflavone) could react with a free radical, by donating a proton, and the resulting quercetin radical formed, possesses low energy to perform further reactions (Mariani et al. 2008) and thus harmful damages (free radical induced) are inhibited. This antioxidant potential of quercetin is mainly due to the hydroxyl (–OH) groups and the catechol B-ring (Rice-Evans et al. 1996, Wang et al. 2006). The arrangement of hydroxyl groups of 3,5,7,3′ and 4′ and a catechol B-ring in quercetin make this compound as a typical antioxidant. Terao and Piskula (1999) have documented that quercetin could interact with phospholipid bilayers and diminish the chain reactive radicals and prevent lipid peroxidation in cell membrane. Quercetin’s free radical scavenging activity was also known to be elevated after chelating magnesium (Mg^2+^) ion (Dehghan and Khoshkam 2012). The antioxidant activity of quercetin could be responsible for the changes in the characteristics of rhythmicities observed in this study.

We observed variations of levels of variables studied over 24-h period. However, many temporal oscillations are insignificant in H_2_O_2_ treated flies. The amplitude values are decreased in this group owing to the non-significant rhythmicities. Temporal oscillation of negative geotaxis assay suggested that H_2_O_2_ treatment caused impairment of this behavior in flies. Elevated production of ROS exhibits a significant role in the modulation of signal transduction pathways and physiological processes of *Drosophila* (Hardeland et al. 2003) and hence variations in negative geotaxis assay may be predicted under oxidative stress. Furthermore, quercetin could reverse the impairment owing to its antioxidant activity. Protein oxidation is characterized by elevated protein carbonyl formation to proteins (Dean et al. 1997). We observed a notable increase in the formation of protein carbonyl content in H_2_O_2_ treated group. The increased generation of free radicals during H_2_O_2_ induced toxicity could cause the elevated production of protein carbonyls and the sequence of formation of protein carbonyl. Rhythmic variation in carbonylated protein content in control flies suggests that a functional clock is involved in the normalization of oxidative injuries (Krishnan et al. 2008). The increased amplitude and mesor of protein carbonyl content in our H_2_O_2_ treated flies indicate the modified circadian regulation owing to oxidative damage in the flies. Similar to our results, impaired temporal pattern of protein carbonyls and products of lipid peroxidation were observed during aging when circadian clock is slowed down in *Drosophila* (Zheng et al. 2007, Krishnan et al. 2009). Quercetin supplementation resulted in a noticeable diminution in the levels of protein carbonyl content. This flavonol could neutralize the free radicals generated during H_2_O_2_ induced oxidative stress and reduced the attack of free radicals on protein. The lipid peroxidation products (TBARS) showed distinct fluctuations over LD cycle in our study, and this could be owing to the homeostatic regulation of the diurnal rhythms of free radical synthesis and antioxidant enzymes (such as SOD and CAT; Subramanian et al. 2014, Seenipandi and Subramanian 2015). The modulated acrophase, amplitude and elevation in mesor of TBARS in group II might be due to the considerable impairment of redox homeostasis by H_2_O_2_ treatment.

The 24 h rhythm of SOD in flies could be due to the synthesis of clock controlled transcription factor, which, could present at high levels in certain circadian phases (Takahashi et al. 2008). Increased mesor values of SOD in quercetin treated flies indicate the reversal towards the normal temporal signature of antioxidant status in the flies. Circadian variations of enzyme activities including CAT have been investigated (Hosamani and Muralidhara 2009) and the peak activity was observed at 13:08 h. The decreased CAT activity could be owing to the higher carbonylation of the enzyme in the flies (Toroser et al. 2007). In addition, since CAT is more susceptible to free radical induced damages than other mitochondrial and peroxisomal proteins (Toroser et al. 2007) the decreased activity is anticipated. Our results corroborate with the previous report that showed peak values of CAT (Hosamani and Muralidhara 2009). The increased mesor values in quercetin treated flies, could be due to, improved levels of antioxidant activity and a rectified or synchronized biological oscillation.

The circadian clock tends to slow down the accrual of products of oxidative damage in organisms by temporally orchestrating the activities of antioxidant enzymes. Genomic microarray studies also showed a synchronous synthesis of several GST enzymes in the fly (Tu and Akgul 2005, Wijnen et al. 2006). Quercetin could positively affect effectiveness of antioxidative defense systems free of its function as a clock component, by performing its function in a pleiotropic noncircadian manner. The mesor values of GST were noticeably elevated in quercetin treated groups which indicate stimulation of GST synthesis. The antioxidant enzyme, GPx, could stop the chain reaction of lipid peroxidation by eliminating lipid peroxides and H_2_O_2_ from membranes (Kinter and Roberts 1996). In our case, the rhythm of GPx showed significant similarity with higher amplitude value in control flies (head and body). Increased amplitude and mesor values in quercetin treated flies might be owing to its potential to elevate glutathione levels.

The non enzymatic antioxidant, GSH, could prevent oxidative damages to cell membrane (Ross 1988) and diminished GSH level is expected after elevation of free radicals in the cells. We also observed an advance in the acrophase of GSH level in H_2_O_2_ treated groups and quercetin supplementation improved the levels of GSH by normalizing the acrophase and amplitude values. Pocernich et al. (2000) reported that increased ingestion of antioxidants, antioxidant precursors or glutathione mimetics could prevent GSH depletion, and thus offer protection in opposition to oxidative stress. Dietary supplementation of antioxidants containing açai and/or resveratrol were known to restore the circadian locomotor activity rhythms in paraquat (an herbicide) treated *D. melanogaster* (Vrailas-Mortimer et al. 2012). The present study demonstrated the tendency towards normalcy in various oxidative stress variables in quercetin treated flies. The insignificant rhythmicity in few indices in head/body indicates tissue level variations in the temporal pattern of H_2_O_2_ susceptibility in the study.

Among numerous Hsps, the stress inducible Hsps 70 kDa (Hsp70) is well-described and investigated for its action during various kind of stresses (Krone et al. 2005). Expression of Hsp70 and other stress proteins could be a biomarker (Gupta et al. 2005, Krone et al. 2005) under stress conditions. It acts as the first bioindicator of cellular damage owing to its conservation from flies to mammals (Krone et al. 2005). In our study the Hsp70 protein expression level was noticed at 04:00 h in WT treated flies (Fig. 2). In *Drosophila* and mammals oxidative stress is usually indicated by the stimulation of Hsp70 genes during aging (Landis et al. 2004). At the time points studied, quercetin is effective almost equally.

IL-6 is a key cytokine with multifold actions and is known to prevent inflammation, uncontrolled cell growth, and immunomodulation in mammals (Beg and Baltimore 1996) in *D. melanogaster* similar roles of Upd1 (homolog of IL-6) have been reported (Amoyel and Bach 2012, Panayidou and Apidianakis 2013). Prior results suggested circadian and even ultradian variations of circulating IL-6 concentrations, with generally lower and higher levels during daytime and night time in humans (Izawa et al. 2013, Vgontzas et al. 2005). Our results also revealed that maximum level of Upd1 expression was at 04:00 in *Drosophila*. Intriguingly, in our study, quercetin possesses anti-inflammatory property and lead to decreased Upd1 expression including at 04:00 wherein the effect of H_2_O_2_ is maximum. NF-κB translocates the transcriptional activators of target genes (Lawrence et al. 2008) including IL-6, iNOS, which play essential roles in preventing oxidative stress mediated reactions and inflammation.

A main consequence of the stimulation of stress-sensitive signaling pathways is the synthesis of nitric oxide which is eventually accountable for the final complications of oxidative stress. Nitric oxide, synthesized by Nos could react with superoxide radical to form the exceedingly reactive radical, peroxynitrite, resulting in more augmented oxidative and nitrosative stress. In the present investigation, upregulation of Nos by quercetin treatment corroborates the results of in other model systems (Cho et al. 2003). The expression of Nos was augmented in H_2_O_2_ treated flies which implies that Nos synthesis may be augmented by the cytokines and/or free radicals formed by H_2_O_2_. However, reduction of Nos expression in H_2_O_2_ with quercetin treated flies may be due to the anti-inflammatory property of quercetin. The modulation of rhythmic expression in quercetin treated group at 04:00 could indicate a stringent regulation of oxidative stress by quercetin.

Differential levels of redox homeostasis have been extensively reported in head and body parts of *D. melanogaster* (Prasad and Muralidhara 2012, 2014). Our results showed dissimilar temporal rhythms of all the variables studied in head and body regions of the fly (Fig. 1, Table 1) and varied responses to quercetin treatment in these organs have also been noticed. To our best knowledge, this is first report showing the differential temporal patterns of the indices of redox homeostasis in head and body regions of the fly.

Our data reveal the efficiency of quercetin in diminishing the harmful influences of H_2_O_2_ toxicity in behavior parameter (geotaxis) together with oxidative stress variables, and its effect on reversing the modulated temporal patterns in *D. melanogaster*. These properties present a mechanism for averting the toxicity evoked by H_2_O_2_. Understanding the underpinnings of the clock in the regulation of redox homeostasis in the amenable system of *Drosophila* will shed light on the working of the behavior and physiology of more complex organisms. From our study however, it is not apparent whether the task of the redox oscillations is to provide an accessory loop or as driver of the clock. Future investigations are desirable in this line to unravel the links between biological clock, redox oscillations and stress inducible proteins.
